# High-yield production of biologically active recombinant protein in shake flask culture by combination of enzyme-based glucose delivery and increased oxygen transfer

**DOI:** 10.1186/1475-2859-10-107

**Published:** 2011-12-12

**Authors:** Kaisa Ukkonen, Antti Vasala, Heikki Ojamo, Peter Neubauer

**Affiliations:** 1 BioSilta Oy, P. O. Box 4300, FI-90014 Oulu, Finland; 2 Bioprocess Engineering Laboratory, Department of Process and Environmental Engineering, University of Oulu, P. O. Box 4300, FI-90014 Oulu, Finland; 3 Chair of Bioprocess Engineering, Department of Biotechnology, Technische Universität Berlin, Ackerstrasse 71-76, D-13355 Berlin, Germany

**Keywords:** Shake flask, Oxygen transfer, Recombinant protein, Fed-batch

## Abstract

This report describes the combined use of an enzyme-based glucose release system (EnBase^®^) and high-aeration shake flask (Ultra Yield Flask™). The benefit of this combination is demonstrated by over 100-fold improvement in the active yield of recombinant alcohol dehydrogenase expressed in *E. coli*. Compared to Terrific Broth and ZYM-5052 autoinduction medium, the EnBase system improved yield mainly through increased productivity per cell. Four-fold increase in oxygen transfer by the Ultra Yield Flask contributed to higher cell density with EnBase but not with the other tested media, and consequently the product yield per ml of EnBase culture was further improved.

## Background

Shake flasks are the most commonly applied laboratory-scale cultivation vessels for production of recombinant proteins due to their very simple set-up and operation. However, on the downside shake flasks do not provide an ideal environment for recombinant protein expression due to relatively low aeration capacity and the batch mode of cultivation. Therefore, the yield of heterologously expressed proteins in shake flasks is usually much lower than in bioreactors where high oxygen transfer rates can be provided and growth rate can be regulated by fed-batch mode of operation based on growth rate limiting glucose feeding.

While it is virtually impossible to apply glucose feeding in a shake flask by an external pump like in a bioreactor, fed-batch-like conditions can be created in simple shaken cultures by an internal glucose release system, called EnBase^® ^technology [1,2]. The EnBase system is based on a soluble polysaccharide in the medium, from which glucose is released through the action of a specific enzyme. In this way, concentration of the enzyme controls the rate at which glucose becomes available in the medium. Enhancement of recombinant protein yield in shaken microbial cultures has been demonstrated with the first generation EnBase system [1], based on a solid starch gel at the bottom of the cultivation vessel, as well as the second generation system [2-5] comprising no solid matrix but a soluble polysaccharide in the liquid medium. The second generation EnBase includes supplementation with complex nutrients, and is therefore not strictly a fed-batch with glucose as the sole available carbon source. The controlled glucose feeding improves recombinant protein expression, especially regarding protein solubility, compared to conventional complex media [2]. The glucose control also enables growth to higher cell densities, which contributes to increased volumetric productivity.

The controlled glucose feeding partly addresses the problem of limited oxygen transfer rate in shake flasks. Growth rate can be adjusted according to the aeration capacity of the vessel, thus preventing the detrimental effects that oxygen limitation may have on cell growth and protein productivity. However, even if EnBase enables aerobic growth at higher cell densities than batch cultures, oxygen will eventually also become limiting when cell density reaches a certain limit. Therefore, growth and protein production in EnBase cultures are expected to be further improved by increased oxygen transfer capacity of the vessel.

**Figure 1:**
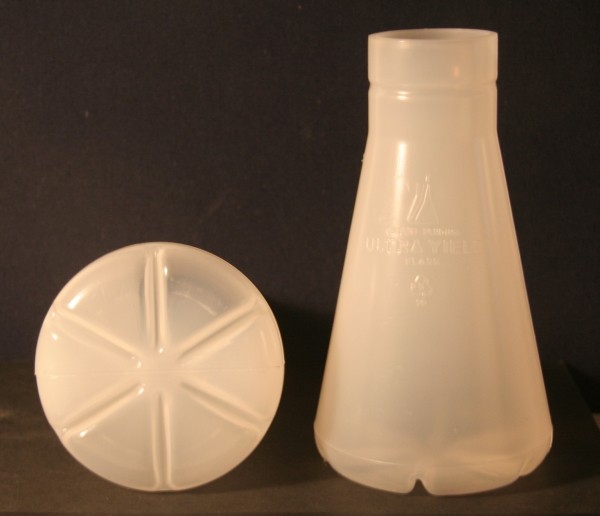
**Ultra Yield Flask with nominal volume of 500 ml**.

A simple approach to improve aeration in shake cultures is to use special flasks such as the plastic Ultra Yield Flask™ (Thomson Instrument Company, USA). These flasks are characterized by a wide neck, steep vertical walls, baffles at the flask bottom, and air-porous membrane closure (Figure 1). They have previously been demonstrated to increase protein yield in Terrific Broth medium [6], mostly through increased cell density while productivity per cell was on average the same as in a baffled Fernbach flask. Another study comparing the Ultra Yield Flask with Fernbach flask also reported increased cell density for 62% of 18 tested *E. coli *clones, though this improvement was obtained only at 18°C and not at 37°C [7].

In this study we demonstrate the combined use of the controlled enzymatic glucose release technology, EnBase, and a high aeration capacity vessel, the Ultra Yield Flask, for high yield production of active recombinant protein. Oxygen transfer coefficients were experimentally determined for the Ultra Yield Flask and a conical glass shake flask, and the influence of the different oxygen transfer capacities on production of correctly folded recombinant protein was evaluated in the EnBase medium, Terrific Broth and ZYM-5052 lactose autoinduction medium.

## Results and Discussion

### Determination of OTR and K_L_a

Oxygen transfer rates (OTR) for 100 ml of aqueous solution were determined in a 500 ml Ultra Yield Flask and 1000 ml conical (Erlenmeyer) glass flask. The applied two-step OTR determination method introduced by Glazyrina et al. [8] allowed for undisturbed shaking of the flasks without any breaks for sample drawing or any measurement devices inside the flask to interfere with the mixing pattern. OTR in the Ultra Yield Flask was calculated to be 118.1 ± 7.3 mmol l^-1 ^h^-1^, corresponding to a K_L_a of 474.1 ± 29.2 h^-1 ^by eq. 4. In the Erlenmeyer OTR and K_L_a were 30.5 ± 4.5 mmol l^-1 ^h^-1 ^and 122.4 ± 18.1 h^-1^, respectively. Hence, OTR and K_L_a in the Ultra Yield Flask were 3.9-fold higher compared to the ordinary Erlenmeyer flask (Table 1). Both flasks were sealed with the same type of seal, the AirOtop Enhanced Seal which is a thin air-permeable membrane keeping the flask sterile but allowing for efficient gas exchange. Therefore, the difference in oxygen transfer between the two flask types arises only from the flask shape and is not affected by differences in flask closure. However, it should be noted that in a typical cultivation shake flasks are often closed with a cotton plug, a foil or metal cap or another type of closure that does not allow for as efficient gas exchange as the AirOtop membrane. Hence OTR and K_L_a determined here for the Erlenmeyer flask are probably significantly higher than in an average shake flask culture with a less permeable closure. This view is supported by the results of Glazyrina et al. [8] who reported almost 10-fold higher K_L_a for Ultra Yield Flask (20% filling volume) compared to Erlenmeyer flask (10% filling volume) although the values for Erlenmeyer were calculated from shaking parameters and not experimentally determined. In the Ultra Yield Flask, the bottom baffles give rise to upward spilling of the liquid, exposing the closure to wetting. However, the AirOtop seals are made of hydrophobic material that enables them to stay dry despite the high degree of liquid spilling. During a long incubation period precipitates of the liquid medium components may accumulate in the membrane and reduce its permeability, but as the flask incubation period in the K_L_a determination experiment was only one hour there were no visible precipitates in the membrane. Therefore, it can be assumed that the permeability of the membrane was not affected by spilling during the K_L_a determination.

**Table 1:** Oxygen transfer rates (OTR) and oxygen transfer coefficients (K_L_a) in 500 ml Ultra Yield Flask and 1000 ml Erlenmeyer flask.

	OTR [mmol l^-1 ^h^-1^]	K_L_a [h^-1^]
**500 ml Ultra Yield Flask**	118.1 ± 7.3	474.1 ± 29.2
**1000 ml Erlenmeyer Flask**	30.5 ± 4.5	122.4 ± 18.1

The measurements were performed for 100 ml of aqueous solution at 30°C with 250 rpm shaking (25 mm offset). Both flask types were sealed with air-permeable membrane seal. The oxygen transfer rates were determined in triplicate measurements and used for calculation of the K_L_a values.

### Use of the EnBase medium and Ultra Yield Flask for recombinant protein production

Heterologous expression of *Lactobacillus *alcohol dehydrogenase (Adh) in *E. coli *RB791 was used as a model system to study the effects of culture medium and flask type on recombinant protein production. Terrific Broth and ZYM-5052 lactose autoinduction medium were used in parallel with the EnBase medium, and cultivations in all three media were performed in both 1000 ml Erlenmeyer flask and 500 ml Ultra Yield (UY) Flask with a broth volume of 100 ml.

**Figure 2:**
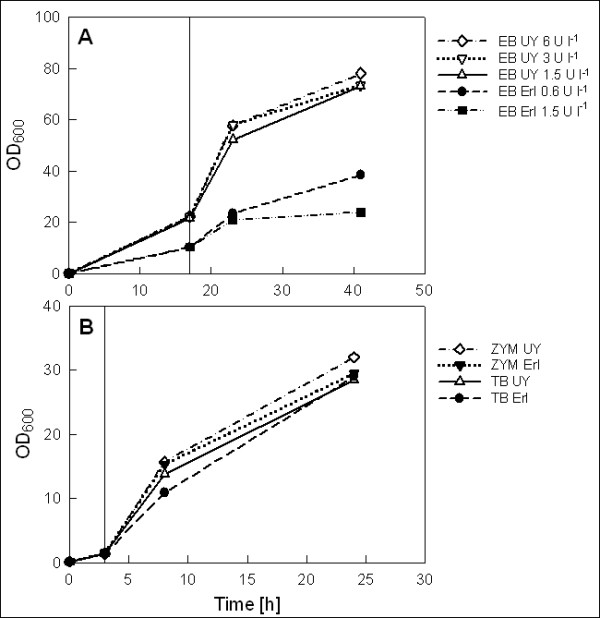
**Cell growth**. Cell density by OD_600 _readings for EnBase (A) as well as TB and ZYM-5052 (B) cultures of *E. coli *RB791[pQE30:adh] in Ultra Yield and Erlenmeyer flasks. One unit of OD_600 _corresponds to dry cell weight of 0.27 g l^-1^. Time of inducer addition is indicated by a straight vertical line. In EnBase cultures the Booster tablet was added at the same time with inducer. Note the different scales in figures A and B. EB: EnBase; TB: Terrific Broth; ZYM: ZYM-5052 autoinduction medium; UY: Ultra Yield Flask; Erl: Erlenmeyer flask. Concentrations of glucose-releasing enzyme in EnBase cultures (A) are presented in the legend in U l^-1^.

**Figure 3:**
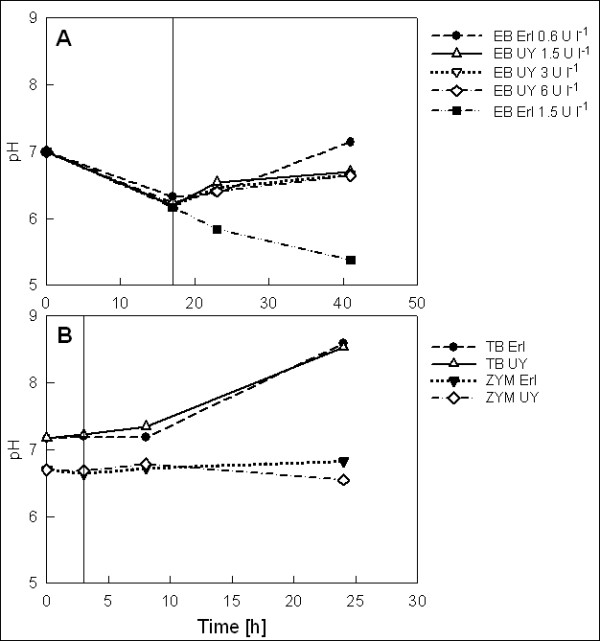
**Medium pH**. pH in EnBase (A) as well as TB and ZYM-5052 (B) cultures of *E. coli *RB791[pQE30:adh] in Ultra Yield and Erlenmeyer flasks. Time of inducer addition is indicated by a straight vertical line. In EnBase cultures the Booster tablet was added at the same time with inducer. Note the different time scales in figures A and B. EB: EnBase; TB: Terrific Broth; ZYM: ZYM-5052 autoinduction medium; UY: Ultra Yield Flask; Erl: Erlenmeyer flask. Concentrations of glucose-releasing enzyme in EnBase cultures (A) are presented in the legend in U l^-1^.

Growth of the cultures was monitored by OD_600 _measurements at induction and at two sampling points after induction (Figure 2). The EnBase cultures were incubated overnight before induction, and OD_600 _at induction was two-fold higher in the UY flask compared to the Erlenmeyer flask despite equal pre-induction concentrations of the glucose-releasing enzyme and inoculum. This suggests that oxygen availability was limiting growth in the Erlenmeyer flask. Final OD_600 _in the UY flasks were 73-78, corresponding to cell dry weight of 19.7-21.1 g l^-1^. Different enzyme concentrations added at the time of induction resulted only in minor differences in cell density. In Erlenmeyer flasks the enzyme concentration had a more profound effect, as addition of 1.5 U l^-1 ^at induction resulted in pH below 6.0 (Figure 3) and consequently a final OD of only 24 (6.5 g l^-1 ^CDW), while the culture with 0.6 U l^-1 ^maintained pH above 6.2 and reached a final OD of 38 (10.3 g l^-1^). The latter is similar to previously reported values for cell yield in the EnBase system in shake flasks [2] as well as in a rocking motion bag bioreactor [9]. The observation that in the UY flasks enzyme addition of even 6 U l^-1 ^at induction did not result in a pH drop below 6.2 (Figure 3) suggests that the low pH in the Erlenmeyer culture with 1.5 U l^-1 ^was not due to glucose overfeeding and acetate overflow but rather due to oxygen limitation and excessive production of anaerobic metabolites. It should also be noted that the higher oxygen availability in the UY Flask enables increased utilization of amino acids as a carbon source through oxidative deamination, resulting in generation of ammonia into the medium. This phenomenon could counteract the medium pH decrease arising from consumption of ammonia and formation of acidic metabolites, making the higher aerated system better buffered against a pH drop. The results clearly demonstrate that compared to ordinary Erlenmeyer flasks the 4-fold higher oxygen transfer in UY flask allows for higher growth rate, higher final cell density and use of higher glucose release rate without causing medium acidification.

Surprisingly, in TB and ZYM-5052 the flask type did not affect either cell density or medium pH (Figure 2, 3). Final OD_600 _of 30 was obtained in both media, corresponding to CDW of 8.1 g l^-1^. This is relatively high compared to cell densities typically obtained in shake flask batch cultures, but might be explained by the choice of media and relatively good aeration also in the Erlenmeyer flasks due to low flask filling volume (10%) and use of the special AirOtop membrane seals. pH in ZYM-5052 was stably maintained at neutral range, while in TB pH increased up to 8.5, indicating high ammonia production due to utilization of amino acids as carbon source.

**Figure 4:**
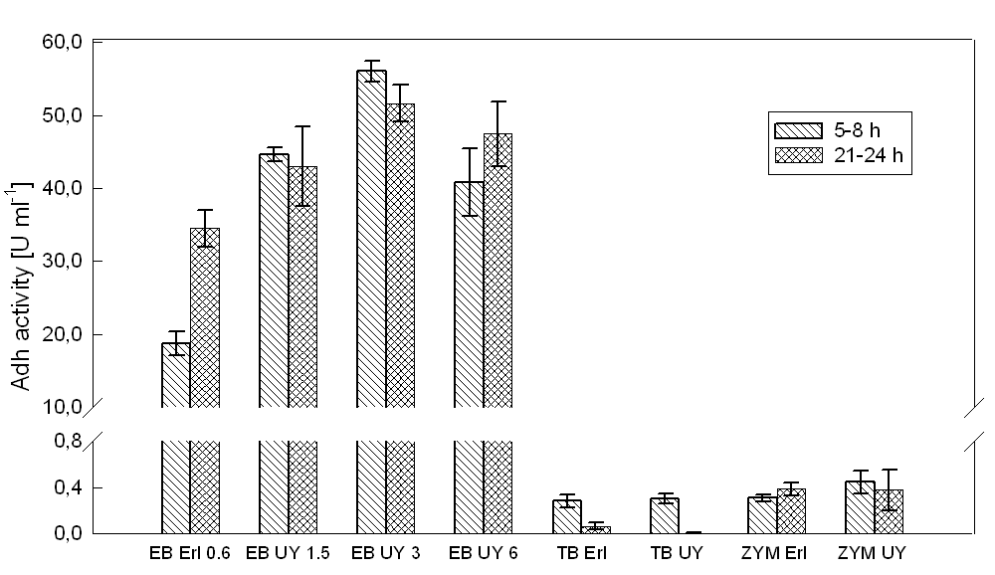
**Adh activity per ml**. Volumetric activity (units per ml of culture broth) of the recombinant Adh product expressed in *E. coli *RB791 in EnBase, TB and ZYM cultures in Ultra Yield and Erlenmeyer flasks. 1 unit is defined as the amount of ADH required for conversion of 1 mM substrate min^-1 ^at 20°C and pH 7.0. EB: EnBase; TB: Terrific Broth; ZYM: ZYM-5052 autoinduction medium; UY: Ultra Yield Flask; Erl: Erlenmeyer flask. The values 0.6, 1.5, 3 and 6 associated with EnBase cultures on the x axis refer to glucose-releasing enzyme concentrations in U l^-1^.

The results for Adh activity in enzyme units per ml of culture broth are presented in Figure 4. Compared to TB and ZYM-5052, the activity yields were very high in EnBase cultures, regardless of flask type. While the highest activities measured for TB and ZYM were 0.30 ± 0.04 U ml^-1 ^and 0.45 ± 0.10 U ml^-1^, respectively, EnBase cultures provided up to 34.5 ± 2.5 U ml^-1 ^in Erlenmeyer and up to 56.1 ± 1.5 U ml^-1 ^in UY flask. Therefore, compared to TB and ZYM-5052 EnBase increased volumetric productivity of active Adh by 113- and 77-fold, respectively, when Erlenmeyer flask was used for cultivation. Through increase in cell density, the higher oxygen transfer in UY flask further improved the volumetric yield of EnBase cultures. Compared to EnBase in the Erlenmeyer flask the increase was up to 1.6-fold. Compared to TB and ZYM-5052, the enhancement by the EnBase and Ultra Yield combination was up to 185- and 125-fold, respectively. In TB and ZYM-5052 the volumetric yield was not affected by the flask type. This finding is in contrast to an earlier study [6], where the authors demonstrated increased yield of 12 different proteins in TB by the UY flask, mainly through increased cell density in the UY flask. In our experiments, high cell density was obtained in TB already in the Erlenmeyer flask, and there was no increase in either the cell or protein yield when UY flasks were used in place of Erlenmeyer flasks.

**Figure 5:**
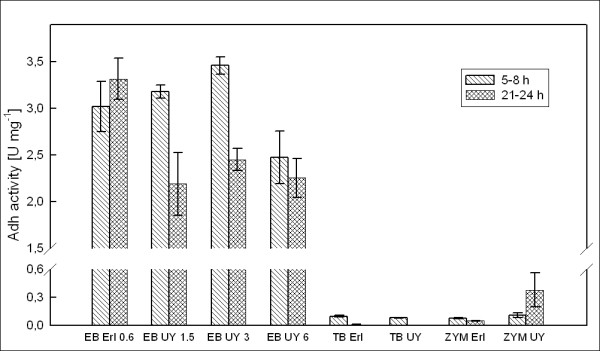
**Adh activity per biomass**. Specific activity (units per mg of cell dry weight) of the recombinant Adh product expressed in *E. coli *RB791 in EnBase, TB and ZYM cultures in Ultra Yield and Erlenmeyer flasks. 1 unit is defined as the amount of ADH required for conversion of 1 mM substrate min^-1 ^at 20°C and pH 7.0. EB: EnBase; TB: Terrific Broth; ZYM: ZYM-5052 autoinduction medium; UY: Ultra Yield Flask; Erl: Erlenmeyer flask. The values 0.6, 1.5, 3 and 6 associated with EnBase cultures on the x axis refer to glucose-releasing enzyme concentrations in U l^-1^.

Specific Adh activity per mg of cell dry weight (Figure 5) was calculated from the volumetric activity data, OD_600 _data and the determined correlation between OD_600 _and CDW. At 6 h after induction, specific productivity of active Adh in the EnBase medium was not significantly influenced by flask type. Therefore it can be concluded that the increase in activity per ml in the UY flask was for the most part due to increase in cell density and not increase in the yield per cell. Compared to the highest measured activity in TB, specific Adh activity was up to 35-fold increased by EnBase in Erlenmeyer flask and up to 36-fold increased by the use of EnBase medium in UY flask. Compared to ZYM-5052, the increase in specific activity was up to 31-fold by EnBase in Erlenmeyer and up to 33-fold by EnBase and UY.

A notable difference between Erlenmeyer and UY flasks was that in Erlenmeyer the specific productivity in EnBase increased slightly from 6 h sample to 24 h sample, while in the EnBase UY flasks specific productivity dropped from 6 h to 24 h by approximately 10-30%. As a result, activity per ml in UY flasks slightly decreased during the extended expression period despite increase in cell density. A possible explanation for the decrease in specific productivity could be exhaustion of ampicillin and consequent overgrowth by plasmid-free non-producing cells in the late phases of EnBase cultivation. The cultivation period in EnBase medium is relatively long (41 h), cell densities are high and no ampicillin was added after the initial dose at inoculation. As the mechanism of ampicillin resistance is secretion of β-lactamase into the extracellular space to degrade ampicillin, high cell densities result in high β-lactamase activity in the medium and the selective pressure will be lost [10]. Therefore, the higher cell density in the UY flasks could result in faster removal of the selective pressure and, consequently, larger fraction of the population being plasmid-free in the end of cultivation. If this is the reason for decreasing activity per cell, the yield in the UY flask might be increased simply by addition of more ampicillin at induction, or by using a vector that applies a more stable antibiotic for selection [11].

**Figure 6:**
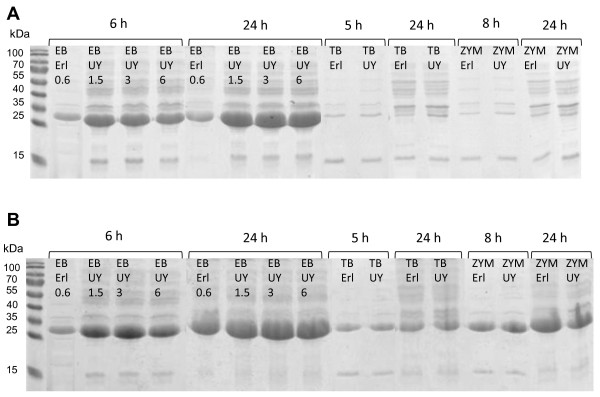
**Cell lysates on reducing SDS-PAGE gels**. A: soluble protein fraction; B: total protein including both soluble and insoluble fractions. The same dilution was used for all samples, i.e. lysates from equal broth volumes were loaded on the gels, and hence the bands represent protein yield per unit volume of culture broth. EB: EnBase; TB: Terrific Broth; ZYM: ZYM-5052 autoinduction medium; UY: Ultra Yield Flask; Erl: Erlenmeyer flask. The values 0.6, 1.5, 3 and 6 associated with EnBase cultures refer to glucose-releasing enzyme concentrations in U l^-1^.

Visualization of total and soluble protein fractions on SDS-PAGE gels (Figure 6) reveals that there was actually relatively high level of Adh expression in TB and ZYM-5052, but that the product was almost exclusively in insoluble form in both the early and late sample. The very low amount of soluble Adh is in agreement with the low level of measured Adh activity in these media. In EnBase cultures most of the expressed Adh was in soluble form as suggested by the virtually equal sizes of total and soluble Adh bands, and the difference in the soluble yield between Ultra Yield and Erlenmeyer flasks is in accordance with the activity data. Similar qualitative difference in the soluble yield between the flask types was also seen for another model protein, recombinant human protein disulfide isomerase, expressed in *E. coli *BL21(DE3) in EnBase medium (data not shown).

The high increase in production of Adh in the soluble and active form by EnBase compared to TB and ZYM-5052 may be at least partly explained by growth rate. In TB and ZYM-5052 Adh expression took place in the exponential growth phase; OD_600 _increased by 10-fold during the 5 h period after induction of TB cultures, corresponding to a doubling time of approximately 1.5 h. In ZYM-5052 growth rate was similar during the same time period. In EnBase cultures, by contrast, doubling time during the first 6 h after induction was approximately 4.5 to 5 h. As suggested previously [2], it is likely that the fast growth in batch media such as TB and ZYM-5052 is associated with a high protein synthesis rate that could result in improper folding and product aggregation, while in EnBase cultures the slower growth allows for slower protein synthesis that may better match the capacity of cellular protein folding machinery. However, a more detailed study on the influence of growth rate would be needed to confirm this. Apart from the growth rate, protein synthesis in TB is likely impeded by the apparently non-optimal ratio of nitrogen compounds and the primary carbon source (glycerol) that results in unfavorably high medium pH through the bacterial metabolism.

In terms of the effect of improved oxygen availability on cell density and volumetric protein yield in the EnBase system, our findings are in good agreement with an earlier report by Pilarek et al. [12] who demonstrated the use of oxygen-saturated liquid perfluorodecalin to enhance oxygen transfer into ml-scale multiwell plate cultures. Introduction of the liquid oxygen carrier into EnBase cultures resulted in 40% higher cell density and correspondingly increased volumetric protein yield in the same Adh-producing clone as used in our study. Therefore, multiwell plates with EnBase and perfluorodecalin constitute an efficient tool for high-throughput screening applications, whereas in shake flask scale the EnBase and Ultra Yield Flask system provides a more cost-efficient alternative to the use of perfluorodecalin or related chemicals. The fed-batch-like glucose control of EnBase provides easy scale-up from shake flask to fed-batch bioreactors [13-15], and the improved oxygen transfer by the Ultra Yield Flask brings the system even closer to the conditions of a stirred bioreactor with high oxygen transfer rate.

## Conclusions

The combination of controlled glucose feeding and a cultivation vessel with highly increased oxygen transfer capacity is a very powerful tool for enhancement of recombinant protein production in simple shaken cultures. Compared to Terrific Broth and ZYM-5052 autoinduction medium, the EnBase medium with the enzymatic glucose release system enhanced the yield of active recombinant Adh by 113- and 77-fold, respectively. Further 1.6-fold improvement in total volumetric yield of EnBase cultures was achieved by the use of Ultra Yield Flask providing 4-fold higher oxygen transfer rate. In this case the higher product yield was due to increased cell density. Due to its very easy operation, the Ultra Yield Flask with the EnBase glucose feeding technology might represent a convenient alternative to laboratory scale bioreactors for high cell density growth and high-yield recombinant protein production, especially in high throughput applications. The fed-batch-like conditions and high oxygen transfer capacity also make the system ideal for process scale-up.

## Methods

### Determination of oxygen transfer coefficients

Oxygen transfer coefficients (K_L_a) were determined for 100 ml of aqueous solution in 500 ml Ultra Yield Flask™ (Thomson Instrument Company, USA) and 1000 ml conical glass flask (standard DIN 12380, ISO 1773). Both flask types were sealed with air-permeable membranes, the AirOtop Enhanced Seals (Thomson Instrument Company, USA). Oxygen transfer rates (OTR) in the flasks were determined in triplicate measurements by an applied sodium sulfite method described in detail by Glazyrina et al. [8], and K_L_a was calculated from the OTR. In brief, 0.25 M sodium sulfite solution was gassed with nitrogen, supplemented with 0.1 μM CoSO_4 _as a catalyst and incubated in a sealed Ultra Yield (UY) or conical flask at 30°C in an orbital shaker with 25 mm offset at 250 rpm for one hour, and the time under shaking was precisely recorded. Immediately after removing the flask from the shaker, the sulfite solution was carefully transferred into a glass beaker. The beaker was stirred with a magnetic stirrer at 23.3 ± 0.7°C. A calibrated oxygen probe was inserted into the beaker to record dissolved oxygen tension (DOT) in 5 min intervals. Stirrer speed, magnet size and the placement and depth of probe insertion in the beaker were always kept the same between measurements. Evaporation from the open beaker was considered to be negligible over the reaction time at room temperature. The DOT data was automatically recorded by MFCS-Win supervisory system (Sartorius, Germany), and the point when sodium sulfite had been completely oxidized to sulfate was observed as a sharp increase in DOT from 0% to 100%. OTR in the shake flask could then be calculated from equation:

(1) $$OTR_{shake\;flask}=\frac{t_{standard}-t_{beaker}}{t_{shake\;flask}}\cdot OTR_{standard}$$

Where t_standard _[h] is the time needed for complete oxidation in the beaker without prior shake flask incubation

t_beaker _[h] is the time needed for complete oxidation in the beaker after shake flask incubation

t_shake flask _[h] is the incubation time in shake flask

OTR_standard _[mmol l^-1 ^h^-1^] is oxygen transfer rate in the beaker.

OTR_standard _was obtained by recording oxidation time in triplicate measurements in the beaker without prior shake flask incubation and inserting the time into equation:

(2) $$OTR_{standard}=\frac{c\left(Na_{2}SO_{3}\right)\cdot v_{O2}}{t_{R}}$$

Where c(Na_2_SO_3_) [mmol l^-1^] is sodium sulfite concentration in the reaction solution

v_O2 _= 0.5, volumetric coefficient for oxygen in the reaction SO_3_^2- ^+ 0.5 O_2 _→ SO_4_^2-^

t_R _[h] is the reaction time needed for complete oxidation.

Finally, the oxygen transfer coefficient K_L_a could be calculated from equation:

(3) $$OTR=K_{L}a\cdot \left(c_{O2}^{*}-c_{O2}\right)$$

Where c_O2_* [mmol l^-1^] is oxygen saturation concentration in the solution

c_O2 _[mmol l^-1^] is oxygen concentration in the liquid phase boundary.

c_O2 _can be assumed to be zero as oxygen is immediately consumed by sulfite oxidation when it enters the liquid phase. Hence K_L_a can be simply obtained from

(4) $$K_{L}a=\frac{OTR}{c_{O2}^{*}}$$

c_O2_* at 30°C was calculated to be 0.249 mmol l^-1 ^by Henry's law:

(5) $$c_{O2}^{*}=\frac{P_{O2}}{H}$$

where P_O2 _is the partial pressure of oxygen (0.21 atm)

H is Henry's law constant; at 30°C, H = 844.32 L · atm · mol^-1 ^[16].

### Bacterial strain

*Lactobacillus *alcohol dehydrogenase (Adh) was heterologously expressed in *Escherichia coli *RB791 [F-, IN(*rrnD*-*rrnE1*), λ-, *lacIqL8*] transformed with plasmid pQE30:adh.

### Media

EnBase medium was constituted by dissolving four EnPresso^® ^medium tablets (BioSilta, Finland) into 100 ml of sterile water. The EnBase medium is composed of a mineral salt medium and phosphate buffer base (for detailed composition of the mineral salt medium see [2]) supplemented with some complex nutrients, trace elements solution and the soluble glucose polysaccharide. The enzyme (*EnZ I'm*; BioSilta) for glucose release from the soluble polysaccharide was added to concentration 0.6 U l^-1 ^shortly before inoculation. At induction the culture was supplemented to a higher concentration of complex nutrients (peptone and yeast extract) by adding the EnPresso Booster tablet.

Terrific Broth (TB) medium contained (per liter): tryptone 12 g; yeast extract 24 g; K_2_HPO_4 _9.4 g; KH_2_PO_4 _2.2 g; 87% glycerol 4 ml.

ZYM-5052 autoinduction medium [17] contained (per liter): tryptone 10 g; yeast extract 5 g; Na_2_HPO_4 _3.56 g; KH_2_PO_4 _3.40 g; NH_4_Cl 2.68 g; Na_2_SO_4 _0.71 g; 87% glycerol 4 ml; glucose 0.5 g; lactose 2 g; trace elements solution 2 ml.

To maintain selective pressure, all media were supplemented with 100 μg ml^-1 ^ampicillin. As high degree of foaming was observed in the UY flasks, Antifoam 204 (Sigma Aldrich) was added to all media in UY flasks at the time of induction.

### Cultivation

*E. coli *RB791[pQE30:adh] was cultivated overnight on Luria-Bertani agar plates with 2 g l^-1 ^glucose and 100 μg ml^-1 ^ampicillin, harvested and stored as a glycerol stock at -70°C. All expression cultures were inoculated with the glycerol stock to OD_600 _of 0.10-0.15. Cultivations were always performed at 30°C in an orbital shaker with 25 mm offset and shaking speed of 250 rpm. Initial broth volume was 100 ml. The cultivation vessels were 500 ml Ultra Yield Flask (Thomson Instrument Company, USA) and 1000 ml conical (Erlenmeyer) glass flask. Both flask types were closed with air-permeable membranes, the AirOtop Enhanced Seals (Thomson Instrument Company, USA). A fresh membrane seal was changed every time the flask was opened for induction or sampling.

The EnBase cultures were induced after overnight cultivation (17 h) with 0.4 mM IPTG. At the same time, two EnPresso Booster tablets (BioSilta) were added to each 100 ml culture together with an additional dose of the *EnZ I'm *(0.6-6 U l^-1^). Samples were harvested for measurement of cell density, pH and product activity at 6 h and 24 h after induction.

Terrific Broth cultures were induced after 3 h incubation at OD_600 _= 1.2-1.5 with 0.4 mM IPTG. Samples were harvested at 5 h and 21 h from induction. Autoinduction cultures were incubated for a total of 24 h, and samples were harvested at 8 h and 24 h.

We have previously observed the optimal time of IPTG addition to be after overnight cultivation (15-18 h) for EnPresso, and the exponential growth phase (OD_600 _= 0.8-1.5) for TB (data not shown). This was the rationale for the different induction times used in these two media. Cultivations were performed in duplicates.

### Analyses

Cell density was recorded by measurement of optical density in 1 ml cuvettes at 600 nm. Cell dry weight (CDW) was determined at the end of cultivation in triplicate samples. The correlation between OD_600 _and CDW was CDW (g l^-1^) = 0.27 · OD_600_.

For pH measurement, 0.2 ml samples were harvested and the cells were spun down. pH was measured from the supernatant with IQ2400 pH probe (IQ Scientific).

For analysis of recombinant protein yield, 100 μl broth samples were centrifuged at 13300 rpm for 4 min at 4°C. Supernatants were discarded and the pellets were frozen at -20°C. After thawing on ice, pellets were resuspended in 100 μl of BugBuster (Novagen). 2 μl of Lysonase Bioprocessing Reagent (Novagen) was added to each sample to lyse the cells. Samples were then centrifuged at 13300 rpm for 4 min at 4°C to remove cell debris.

Proteins in the cell lysate were visualized on reducing SDS-PAGE gels stained with Coomassie Brilliant Blue. Total proteins (insoluble and soluble fraction) were analyzed from the lysates before centrifugation, and soluble protein fractions were analyzed from the lysate supernatant after removal of debris and insolubles by centrifugation.

Adh activity was determined by the following assay: the lysate supernatants were sufficiently diluted in triethanolamine buffer (100 mM triethanolamine, 1 mM MgCl_2_, pH 7.0). 2 μl of the diluted sample was added to a polystyrene microwell plate (Greiner), and 218 μl of substrate solution (100 mM triethanolamine, 1 mM MgCl_2_, 18.4 mM ethyl-4-chloroacetoacetate, 147 μg ml^-1 ^NADPH) was added to the well. Absorbance at 340 nm was recorded every 10 s for 5 min period. Every sample was measured in triplicate. The absorbance readings were plotted against time, and slope (ΔE min^-1^) was determined for the initial linear part of the curve. Activity of Adh in the sample was calculated from equation:

(6) $$Acitivity\;\left(U\;ml^{-1}\right)=\frac{\Delta E\;min^{-1}\cdot X\cdot V_{total}}{\varepsilon_{NADPH}\cdot V_{sample}\cdot d}$$

where X is the dilution factor

V_total _[ml] is the total volume of reaction mixture in the well

ε_NADPH _is extinction coefficient for NADPH (6.22 mM^-1 ^cm^-1^)

V_sample _[ml] is sample volume

d [cm] is the light path (sample height)

One unit (U) of Adh activity is here defined as the amount of Adh required for conversion of 1 mM substrate (ethyl-4-chloroacetoacetate) min^-1 ^at 20°C and pH 7.0.

## Competing interests

The authors declare that they have no competing interests.

## Authors' contributions

KU designed and conducted all experiments and prepared the manuscript. AV participated in the design of cultivation experiments, data analysis and manuscript preparation. HO assisted in designing the K_L_a measurements and manuscript preparation. PN participated in the study design, data analysis and manuscript preparation. All authors read and approved the final manuscript.
